# Knowledge about permanent tooth avulsion and its management among dentists in Riyadh, Saudi Arabia

**DOI:** 10.1186/s12903-015-0126-3

**Published:** 2015-11-02

**Authors:** Yousra Hussain AlJazairy, Hassan Suliman Halawany, Nassr AlMaflehi, Nawaf Sulaiman Alhussainan, Nimmi Biju Abraham, Vimal Jacob

**Affiliations:** Department of Restorative Dentistry, College of Dentistry, King Saud University, Riyadh, Kingdom of Saudi Arabia; Dental Caries Research Chair, College of Dentistry, King Saud University, Riyadh, Kingdom of Saudi Arabia; Department of Periodontics and Community Dentistry, College of Dentistry, King Saud University, Riyadh, Kingdom of Saudi Arabia; Dental Health Department, Prince Sultan Military Medical City, Riyadh, Kingdom of Saudi Arabia

**Keywords:** Knowledge, Avulsion, Dentists

## Abstract

**Background:**

There is a lack of adequate information on dentists’ knowledge about tooth avulsion and its management in Saudi Arabia. The aim of this study was to evaluate the level of knowledge about permanent tooth avulsion and its management among dentists working in Riyadh, Saudi Arabia.

**Methods:**

A total of 550 dentists were contacted to request their participation in this 19-item questionnaire survey over a three-month period starting in January 2015 using convenience sampling methodology. A questionnaire consisting of demographic items and multiple-choice questions regarding knowledge of avulsion and its management was used. The level of knowledge was assessed using a scoring system that assigned one point for each correct answer and zero points for wrong answers, with a maximum possible score of ten points. An independent t-test was used to compare the level of knowledge (mean score) based on particular variables, such as sex, nationality, type of practice, current employment, working hours and whether the respondents had attended a continuing dental education (CDE) programme on avulsion management. The level of significance was set at *P*< 0.05.

**Results:**

A total of 470 completed questionnaires were collected with data suitable for statistical analysis. The majority of the respondents were Saudi (*n =* 331; 72.1 %) and general practitioners (*n =* 278; 59.3 %). Most of the participants correctly responded to the knowledge-based questions, except the questions regarding the best storage medium (milk: 24.1 %) and the duration of follow-up by clinical and radiographic examination (5 years: 15.6 %). The mean knowledge score was 5.94 ± 1.57. Gender (*P =* 0.001), current employment (*P =* 0.045) and working hours per day (*P =* 0.020) had a significant effect on the mean knowledge score.

**Conclusions:**

The surveyed dentists were found to have a moderate knowledge of avulsion and its management, although a relative lack of knowledge was observed regarding the duration of follow-up after replantation.

## Background

Permanent tooth avulsion occurs in 0.5–3 % of all dental injuries [1], which are reportedly the most common cause of all cases of facial trauma. The prognosis of an avulsed tooth is essentially dependent on its extra-alveolar time and the procedures performed at the time of the avulsion injury. In cases where these factors are unfavourable, pulp necrosis and degeneration of periodontal ligament (PDL) cells may ensue, resulting in inflammatory/replacement root resorption or ankylosis of the tooth, eventually leading to tooth loss [1, 2]. Immediate replantation is the treatment of choice for an avulsed tooth, although it is not always possible to perform this treatment [1]. Replantation success depends on the maintenance of PDL cell vitality [3].

A higher prevalence of dental trauma in 12- to 14-year-old boys (prevalence of avulsion: 6.4 per thousand permanent maxillary central incisors) was reported in a Saudi Arabian study [4] compared with that found in an age-matched United Kingdom Children’s Dental Health Survey (prevalence of avulsion: 3.0 and 0.8 per thousand permanent maxillary central incisors for 12- and 14-year-old boys, respectively) [5]. A considerably higher prevalence of maxillary central incisor trauma (31.4 %) was reported among 12- to 15-year-old Saudi girls in another study [6].

Comparatively few Saudi studies have investigated knowledge of tooth avulsion and its management across various professions that are either directly or indirectly involved in emergency dental trauma care. A recent survey performed by Halawany et al. [7] reported that the educational qualifications of dental assistants had a significant effect on their level of knowledge regarding tooth avulsion and its management. However, there is a lack of sufficient information regarding dentists’ knowledge about tooth avulsion and its management in Saudi Arabia. Consequently, the aim of this study was to evaluate the knowledge of dentists working in private and governmental hospitals, clinics, polyclinics (large dental health care facilities that provide a wider range of service than a standard general dental practitioner’s office) and dental schools in Riyadh, Saudi Arabia, concerning permanent tooth avulsion and its management.

## Methods

The current study was registered with and approved by the College of Dentistry Research Center (CDRC: registration number FR 0211), and informed written consent was provided by each subject according to the ethical principles of the World Medical Association Declaration [8]. A 19-item English-language questionnaire, which was a modified version of the questionnaire used by Westphalen et al. [9], was developed and pretested in a group of ten Saudi and non-Saudi dentists. Potential difficulties were identified regarding the respondents’ comprehension of the questionnaire, which consisted of demographic items and multiple-choice questions regarding dental trauma and its management, and minor modifications were made according to these results. The final questionnaire consisted of 9 demographic items and 10 multiple-choice questions regarding dental trauma and its management. The survey was administered to dentists working in various private and governmental hospitals, clinics, polyclinics and dental schools in Riyadh, Saudi Arabia.

According to the Ministry of Health Statistics 2014, the total number of dentists working in Riyadh is 3597, of which 2929 work in the private sector, and 668 work in the government sector [10]. A total of 550 dentists, representing 15.3 % of all dentists working in Riyadh, were contacted to request their participation in this study over a period of three months starting in January 2015 using convenience sampling methodology. Two of the co-investigators participated in the data collection process and approached each of the prospective participants individually in person. The dentists’ willingness to participate in the study by completing the anonymous questionnaire was sought. No personal information on the dentists’ identities was required to be disclosed. Dentists who were willing to participate were given the questionnaire, and they completed and returned the questionnaire immediately to the co-investigators.

The data obtained from the survey were manually entered into a Statistical Package for the Social Sciences database (IBM, SPSS version 20, IL, USA) and analysed with a significance level established at *P <* 0.05. The respondents’ level of knowledge regarding tooth avulsion and its management was assessed using a scoring system that assigned one point for each correct answer and zero points for wrong answers, with a maximum possible score of ten points. An independent t-test was used to compare the level of knowledge (mean score) based on variables such as sex, nationality, type of practice, current employment, working hours, and whether the respondents had attended a continuing dental education (CDE) programme on avulsion management. Logistic regression was performed to assess the significance of each demographic variable (sex, nationality, type of practice, years of experience, current employment and working hours) and CDE programme attendance on avulsion management to explain their likelihood of correctly answering each of the knowledge-based questions.

## Results

A total of 470 dentists, representing 13.1 % of the dentists working in Riyadh, completed the questionnaires with data that were suitable for statistical analysis. The mean age of the respondents was 35.89 ± 9.09 years. The majority of the respondents were Saudi (*n =* 331; 72.1 %) and general practitioners (*n =* 278; 59.3 %). The demographic characteristics of the study population are shown in Table 1.

**Table 1:** Demographic characteristics of the study population

Variables		Total *N =* 470, n (%)
Sex^a^	Male	220 (46.9)
	Female	249 (53.1)
Nationality^b^	Saudi	331 (72.1)
	Non-Saudi	128 (27.9)
Educational qualifications^c^	Bachelor’s Degree	269 (57.2)
	Master’s Degree	174 (37.0)
	Doctorate	24 (5.8)
Type of practice	General Practitioners	278 (59.3)
	Endodontists	50 (10.6)
	Orthodontists	35 (7.4)
	Pedodontists	26 (5.5)
	Oral and Maxillofacial Surgeons	21 (4.5)
	Prosthodontists	18 (3.8)
	Periodontists	16 (3.4)
	Dental Public Health Specialists	11 (2.3)
	Advanced General Dentists	6 (1.3)
	Oral Biologists	4 (0.9)
	Oral Medicine Specialists	3 (0.6)
	Oral Pathologists	2 (0.4)
Years of experience^d^	<5 years	287 (62.5)
	6 to 15 years	92 (20.0)
	>16 years	80 (17.5)
Current employment^e^	Private	138 (30.5)
	Public	315 (69.5)
Working hours^f^	≤8 h	299 (66.6)
	>8 h	150 (33.4)
Attended continuing dental education programme on the management of tooth avulsion^g^	Yes	195 (41.7)
	No	273 (58.3)

Missing values: a = 1; b = 11; c = 3; d = 11; e = 17; f = 21; g = 2

The percentage distribution of the responses to the questions regarding avulsion knowledge and management is shown in Table 2. Most of the participants correctly responded to the knowledge-based questions, except those regarding the best storage medium and the duration of follow-up by clinical and radiographic examination. Questions regarding factors that may affect the outcome of replantation of an avulsed tooth were correctly answered by the highest number of respondents (*n =* 415; 89.4 %), followed by questions regarding splinting duration (*n =* 389; 83.5 %).

**Table 2:** Distribution of the responses to questions about avulsion and its management

Questions about avulsion and its management		*n* (%)
1) Should an avulsed permanent tooth be replaced in its socket?^a^	Yes, in all cases	105 (22.6)
	Not in all cases^k^	354 (76.3)
	Never	5 (1.1)
2) Factors that may influence the outcome of replantation of the avulsed tooth^b^	Extra-alveolar period	23 (5.0)
	Storage medium	20 (4.3)
	Injury to periodontal ligament	6 (1.3)
	All of the above^k^	415 (89.4)
3) Best storage medium^c^	Patient’s saliva	121 (26.1)
	Milk^k^	112 (24.1)
	Physiological saline solution	22 (4.8)
	Hank’s balanced salt solution	209 (45.0)
4) Ideal extra-alveolar period^d^	<30 min^k^	318 (68.5)
	30 minutes to 1 h	127 (27.4)
	1 to 2 h	19 (4.1)
5) Tooth management before replantation^e^	Hold the crown and wash with any antiseptic solution	24 (5.2)
	Hold the crown and wash with physiological saline solution^k^	329 (70.9)
	Hold the crown and wash with tap water	73 (15.7)
	Hold the root and wash with physiological saline solution	38 (8.2)
6) Type of splinting^f^	Flexible splints^k^	240 (51.6)
	Rigid splints	215 (46.2)
	No need for splinting	10 (2.2)
7) Splinting duration^g^	Less than 7 days	33 (7.1)
	7 to 14 days^k^	389 (83.5)
	30 days	44 (9.4)
8) Endodontic treatment^h^	Pulpectomy and root canal filling after 15 days	119 (25.6)
	Depends on extra-alveolar period and stage of root formation^k^	287 (61.7)
	Immediate pulpectomy and calcium hydroxide therapy	59 (12.7)
9) Systemic medication^i^	Prescribe anti-inflammatory drugs only	101 (21.7)
	Prescribe antibiotics, anti-inflammatory drugs and tetanus prevention^k^	274 (59.1)
	No medication required	89 (19.2)
10) Follow-up by clinical and radiographic examination for:^j^	1 year	280 (59.8)
	3 years	115 (24.6)
	5 years^k^	73 (15.6)

Missing values: a = 6; b = 6; c = 6; d = 6; e = 6; f = 5; g = 4; h = 5; i = 6; j = 2

^k^Best answer from the choices provided

The mean knowledge score regarding tooth avulsion and its management according to demographic variables is shown in Table 3. The overall mean score was 5.94 ± 1.57. The mean knowledge score of female participants was significantly higher than that of male participants (*P =* 0.001). Furthermore, a significantly higher mean knowledge score was observed among respondents working in public clinics/hospitals compared with those working in private clinics/hospitals (*P =* 0.045) and among respondents working 8 h or fewer per day compared with those working more than 8 h per day (*P =* 0.020).

**Table 3:** Mean knowledge scores regarding avulsion and its management according to demographic variables

Demographic variable		Mean ± SD	*P*-value
Sex	Male	5.68 ± 1.54	0.001^a^
	Female	6.17 ± 1.56	
Nationality	Saudi	5.99 ± 1.53	0.648
	Non-Saudi	5.91 ± 1.60	
Type of practice	General practitioner	5.92 ± 1.44	0.783
	Specialist	5.96 ± 1.77	
Current employment	Private	5.72 ± 1.54	0.045^a^
	Public	6.05 ± 1.59	
Working hours	≤8 h	6.06 ± 1.52	0.020^a^
	>8 h	5.69 ± 1.65	
Attended continuing dental education programme on tooth avulsion management	Yes	5.89 ± 1.62	0.648
	No	5.96 ± 1.53	

Statistical test: Independent T test

^a^Significant

Logistic regression analyses for each of the knowledge-based questions according to the background characteristics of the respondents are shown in Table 4. The likelihood of correctly answering most of the knowledge-based questions was significantly higher among respondents working in public clinics/hospitals (7 of 10 questions) compared with those working in private clinics/hospitals, followed by female participants (6 of 10 questions) compared with male participants.

**Table 4:** Odds ratio based on maximum-likelihood estimates using logistic regression

Qs^a^	Sex (1)	Nat (1)	Prac (1)	Emp (1)	Hrs (1)	CDE (1)
1	**1.90**	**1.77**	1.03	**3.07**	0.76	0.67
	(1.29–2.82)	(1.04–3.04)	(0.56–1.92)	(2.04–4.63)	(0.47–1.23)	(0.43–1.04)
2	**2.71**	**2.49**	0.95	**3.81**	1.03	1.95
	(1.59–4.61)	(1.15–5.41)	(0.41–2.19)	(2.29–6.36)	(0.53–2.01)	(1.04–3.65)
3	0.95	0.59	0.55	**0.36**	0.72	0.86
	(0.64–1.42)	(0.33–1.04)	(0.28–1.08)	(0.24–0.55)	(0.42–1.21)	(0.54–1.37)
4	**1.61**	1.52	1.65	**1.66**	0.63	0.82
	(1.11–2.34)	(0.90–2.55)	(0.91–3.00)	(1.14–2.42)	(0.40–1.00)	(0.54–1.25)
5	1.27	**1.78**	1.22	**3.05**	1.03	0.66
	(0.87–1.85)	(1.06–3.01)	(0.67–2.24)	(2.03–4.56)	(0.64–1.65)	(0.43–1.01)
6	1.18	0.75	**2.09**	0.99	0.65	**1.68**
	(0.83–1.68)	(0.47–1.20)	(1.19–3.68)	(0.69–1.43)	(0.42–1.01)	(1.13–2.52)
7	**2.56**	**1.92**	0.66	**2.61**	1.11	**3.44**
	(1.61–4.09)	(1.03–3.59)	(0.32–1.33)	(1.66–4.12)	(0.63–1.96)	(1.92–6.16)
8	**1.67**	**0.59**	0.79	1.38	0.86	1.26
	(1.17–2.39)	(0.37–0.95)	(0.45–1.38)	(0.96–1.98)	(0.56–1.34)	(0.84–1.88)
9	1.37	1.32	1.07	1.25	1.01	0.88
	(0.96–1.95)	(0.81–2.15)	(0.61–1.86)	(0.87–1.80)	(0.65–1.58)	(0.59–1.32)
10	**0.51**	0.69	**2.19**	**0.30**	0.62	**0.45**
	(0.33–0.80)	(0.40–1.26)	(1.09–4.36)	(0.19–0.47)	(0.34–1.12)	(0.26–0.77)

Confidence interval (lower–upper) in parentheses. The variables with bold numbers were statistically significant (*P <* 0.05)

^a^Questions 1 to 10 are provided in Table 2

Sex: (0) – Male, (1) – Female; Nationality (Nat): (0) – Saudi, (1) – Non-Saudi; Type of practice (Prac): (0) – General practitioner, (1) – Specialist; Current employment (Emp): (0) – Private, (1) – Public; Working hours (Hrs): (0) – ≤ 8 h, (1) – > 8 h; Attended continuing dental education programme on avulsion management (CDE): (0) – No, (1) – Yes

## Discussion

Knowledge of avulsion, which is considered a dental emergency, and its management can reduce stress and anxiety for both patients and dentists [11]. Correct immediate post-traumatic management protocols have been reported to improve both the short- and long-term prognosis of the avulsed tooth [12]. Several studies have assessed dental trauma knowledge among dentists and reported that the surveyed dentists had insufficient knowledge to treat dental trauma [13–15], had very little experience treating dental trauma to permanent incisors [16], and had a lack of confidence regarding the management of complex trauma cases [17]. The purpose of our cross-sectional study was to evaluate the level of knowledge about avulsion and its management among dentists working in Riyadh, Saudi Arabia.

### Demographics

The level of knowledge of the surveyed sample of dentists was found to be moderate, with an overall mean knowledge score of 5.94 ± 1.57. The results of our study also revealed that gender, current employment and daily working hours had a significant effect on the mean knowledge score, whereas nationality, type of practice and history of attending CDE programmes on the management of tooth avulsion had no significant effect (Table 3). These findings were not consistent with the results obtained in previous studies [14, 18]; however, these studies used different questions to determine the knowledge score.

Most of our respondents (58.3 %) reported that they had not attended any CDE programme on tooth avulsion management, which is consistent with the findings reported by Zhao et al. [19] but not with those reported in other studies [9, 14]. Most of the respondents (60 %) in the study by Westphalen et al. [9] reported that they had participated in a CDE programme on dental trauma on their own initiative after graduation. The authors stated that the city where the study was performed had four dental schools; thus, the local dental practitioners had a higher probability of being presented with opportunities to attend these courses. Although Riyadh is the venue for the annual Saudi Dental Society International Dental Conference, and there are eight public and private dental schools within the city, fewer respondents had attended CDE programmes on this vital aspect of emergency dental care and management. This finding may be due to a lack of related course offerings, a lack of interest, or busy work schedules among these dentists. However, no significant difference (*P =* 0.648) in the mean knowledge score was observed between those who had and had not attended a CDE programme (Table 3). This result is not consistent with previous studies, which reported that dentists who attended dental trauma courses after graduation had higher knowledge scores [13], had more thorough [15] and better [14] knowledge of dental trauma management and had more confidence in managing these patients [17]. Some of the respondents who reported having attended CDE programmes on this topic may have done so a long time ago, and the information provided may now be outdated. This is a potential reason for the lack of a significant difference in the mean knowledge score between those who had and had not attended a CDE programme.

### Clinical management

Approximately 76 % of our respondents reported that an avulsed permanent tooth should not be replanted in all cases, which is consistent with the results of previous studies [19, 20]. In cases of severe caries, periodontal disease, and medical conditions, such as immunosuppression or severe cardiac diseases, or in cases in which the patient is not cooperative, replantation of the avulsed permanent tooth is not indicated according to the International Association of Dental Traumatology (IADT) guidelines [1]. Most of our respondents (89.4 %) reported that the extra-oral period, storage medium and injury to the PDL are factors that may affect the outcome of replantation of the avulsed tooth, which is consistent with the results of a study by Westphalen et al. [9]. The extra-alveolar period has been recognized as the most critical factor for optimal periodontal healing [21, 22].

Saline, Hank’s balanced salt solution (HBSS), and milk are examples of osmolality-balanced media suitable for storing avulsed teeth [1]. The patient’s saliva, although readily available at the site of trauma, contains bacteria and their by-products [23]. Furthermore, several studies have reported that the vitality of PDL cells can be sustained for 30 min when immersed in the patient’s saliva, but it decreases remarkably after 60 min [21, 23, 24]. However, while milk may not be readily available at the site of trauma, storage of the avulsed tooth in milk at room temperature has been reported to preserve the viability of PDL cells for up to 60 min, whereas refrigerated milk preserves viability for an additional 45 min [25, 26]. HBSS, on the other hand, was not included by some authors as an option in their questionnaire concerning storage medium due to its lack of availability at trauma sites [27]. Physiological saline solution is more commonly available than HBSS but is less available than milk. Thus, for the purpose of scoring in this study, one point was assigned to the respondents who identified milk as the best storage medium. The highest percentage of our respondents (45 %) reported HBSS as the best storage medium, followed by the patient’s saliva (26.1 %) and milk (24.1 %). This finding was not consistent with the results of previous studies, in which most of the participants reported the patient’s saliva [9], saline [18], or milk [20, 28, 29] as the preferable or recommended storage medium.

The highest percentage of participants (68.5 %) reported the ideal extra-alveolar period as less than 30 min, which is consistent with the results of previous studies [9, 13, 19, 20]. Furthermore, approximately 71 % of our respondents reported holding the crown of the avulsed tooth and washing it with physiological saline solution prior to replantation, and 15.7 % of the respondents reported holding the crown and washing it with tap water. Westphalen et al. [9] reported that a significant number of the participating dentists in their study (40 %) used tap water to wash the avulsed tooth prior to replanting it during an office or hospital procedure. However, Baginska et al. [18] reported that most of their respondents (85 %) treated the contaminated avulsed tooth by gently rinsing it with saline under dental surgical conditions. The IADT recommends briefly washing the avulsed tooth (for a maximum of 10 s) under cold running water before repositioning it in the alveolar socket as a first-aid procedure at the accident site and cleaning the root surface and apical foramen with a stream of saline in cases in which the avulsed tooth has been kept in a physiological storage medium and/or the stored dry and extra-oral dry time was less than 60 min [1]. Because our questionnaire did not specify the clinical conditions (i.e., whether the procedures were performed at the accident site or whether the avulsed tooth was brought to the dental office in a suitable storage medium within 60 min), the results of this questionnaire should be cautiously interpreted.

The IADT recommends using a flexible splint for up to 14 days for an avulsed tooth [1]. Although more of our respondents suggested a flexible splint, the difference in the number of respondents suggesting flexible (51.6 %) compared with rigid (46.6 %) splints was not marked. However, a significantly higher number of respondents (83.5 %) reported the duration of splinting as 7 to 14 days. These results were not consistent with those obtained in previous studies. Hu et al. [14] reported that only 59.1 % of their participants knew that a flexible splint is indicated for 2 weeks in cases of avulsed teeth. Westphalen et al. [9] reported that most of their respondents (73 %) suggested a flexible splint and that 64 % of the dentists reported a splinting duration of more than 15 days. However, Zhao et al. [19] reported that more of their respondents suggested a rigid splint (49 %) than a flexible splint (45.1 %). Furthermore, a higher percentage of the respondents in this previous study suggested splinting for 30 days (40.6 %), whereas only 10.2 % suggested splinting for 2 weeks.

The IADT recommends root canal treatment if the dry time exceeds 60 min or for other reasons, such as the presence of non-viable cells and teeth with a closed apex [1]. A large percentage of our respondents (61.7 %) reported that the endodontic treatment is dependent on the extra-alveolar period and stage of root formation, which is consistent with the results obtained by Westphalen et al. [9]. Most participants in the study by Krastl et al. [27] reported that root canal treatment should be performed within 7 to 14 days for an avulsed tooth (with completed root apex formation) that has been replanted within 30 min. The IADT recommends prescribing systemic antibiotics and referring the patient to a physician to evaluate the need for a tetanus booster in cases in which the avulsed tooth has contacted soil or the tetanus coverage is uncertain [1]. A higher percentage of our respondents (59.1 %) suggested prescribing antibiotics, prescribing anti-inflammatory drugs and employing tetanus prevention strategies, which was consistent with the results obtained by Westphalen et al. [9]. Antibiotic treatment after replantation was also recommended by most of the participants in the study by de Vasconcellos et al. [28].

### Follow-up

Follow-up treatment of replanted teeth via clinical and radiographic examination is recommended for a period of 5 years [22, 30]. However, more of our respondents suggested follow-up treatment by clinical and radiographic examination for 1 year (59.8 %), which was not consistent with the results of the study by de Vasconcellos et al. [28]. The highest percentage of the participants in that study (34.8 %) stated that the correct practice was follow-up treatment by clinical and radiographic examination for 2 or more years.

### Study limitations

Specific limitations of this study should be noted when interpreting the results. A major limitation is the sampling methodology implemented in our study. A convenience sample may not sufficiently represent the entire population of dentists working in Riyadh. A direct comparison of our results with those of previous studies was not always possible due to differences in the questions and answer choices. Several studies used multiple-choice questions [18–20, 28], open-ended questions [9] or scenario-based questions [14]. Furthermore, some studies determined the frequency distribution of the participants according to the questions [9, 20], whereas other studies used a scoring system [14, 18]. The basis for selecting the best storage medium (availability and maintaining the viability of PDL cells) was not explained in our questionnaire, which may have resulted in a higher number of respondents selecting HBSS instead of milk. The cross-sectional study design and lack of a control group should also be considered limitations.

## Conclusions

Within the limitations of this study, the surveyed dentists’ knowledge regarding avulsion and its management was found to be moderate, although a relative lack of knowledge was observed regarding the duration of follow-up after replantation. While most of the respondents correctly answered most of the questions according to the IADT guidelines, a small but significant number of respondents answered the questions incorrectly. Future studies using clinical scenario-based questions related to permanent tooth avulsion and its management will enable more robust testing of dentists’ knowledge.

## Abbreviations

PDL: Periodontal ligament
CDRC: College of Dentistry Research Center
SPSS: Statistical Package for the Social Sciences
CDE: Continuing dental education
ANOVA: Analysis of variance
IADT: International Association of Dental Traumatology
HBSS: Hank’s balanced salt solution
